# Growth of Anodic Layers on 304L Stainless Steel Using Fluoride Free Electrolytes and Their Electrochemical Behavior in Chloride Solution

**DOI:** 10.3390/ma15051892

**Published:** 2022-03-03

**Authors:** Laura Patricia Domínguez-Jaimes, María A. Arenas, Ana Conde, Beatriz Escobar-Morales, Anabel Álvarez-Méndez, Juan Manuel Hernández-López

**Affiliations:** 1 Facultad de Ciencias Químicas, Universidad Autónoma de Nuevo León, Avenida Pedro de Alba s/n, San Nicolás de los Garza 66455, Nuevo León, Mexico; laura.dominguezjms@uanl.edu.mx (L.P.D.-J.); anabel.alvarezmn@uanl.edu.mx (A.Á.-M.); 2 Department of Surface Engineering Corrosion and Durability, National Center for Metallurgical Research, CENIM-CSIC, Avda. Gregorio del Amo, 8, 28040 Madrid, Spain; geles@cenim.csic.es (M.A.A.); a.conde@cenim.csic.es (A.C.); 3 CONACYT—Centro de Investigación Científica de Yucatán, Carretera Sierra Papacal-Chuburná Puerto, Km 5, Mérida 97302, Yucatán, Mexico; beatriz.escobar@cicy.mx

**Keywords:** anodizing, fluoride-free electrolyte, stainless steel, corrosion resistance

## Abstract

Anodic layers have been grown on 304L stainless steel (304L SS) using two kinds of fluoride-free organic electrolytes. The replacement of NH_4_F for NaAlO_2_ or Na_2_SiO_3_ in the glycerol solution and the influence of the H_2_O concentration have been examined. The obtained anodic layers were characterized by scanning electron microscopy (SEM), energy-dispersive X-ray spectroscopy (EDX), X-ray photoelectron spectroscopy (XPS), Raman spectroscopy, and potentiodynamic polarization tests. Here, it was found that, although the anodic layers fabricated within the NaAlO_2_-electrolyte and high H_2_O concentrations presented limited adherence to the substrate, the anodizing in the Na_2_SiO_3_-electrolyte and low H_2_O concentrations allowed the growth oxide layers, and even a type of ordered morphology was observed. Furthermore, the electrochemical tests in chloride solution determined low chemical stability and active behavior of oxide layers grown in NaAlO_2_-electrolyte. In contrast, the corrosion resistance was improved approximately one order of magnitude compared to the non-anodized 304L SS substrate for the anodizing treatment in glycerol, 0.05 M Na_2_SiO_3_, and 1.7 vol% H_2_O at 20 mA/cm^2^ for 6 min. Thus, this anodizing condition offers insight into the sustainable growth of oxide layers with potential anti-corrosion properties.

## 1. Introduction

Significant progress has been reached in nanoscience and nanotechnology fields to improve the properties, performance, and durability of materials. Mainly, surface and interface engineering has developed surface properties in metallic materials [1].

One of the most common surface modification methods is the anodizing process, which provides a unique combination between functionality and surface morphology, different from bulk material [2]. During the anodizing, a target metal is connected to the positive terminal of a power supply unit (anode) and a platinum or very stable metal to the negative terminal of a power supply (cathode), and a direct current is applied to an electrolyte [3]. Due to the applied stimulus, the metal is oxidized. It generates metal cations that migrate outward up to the metal/electrolyte interface, which react with the anions (e.g., O_2_^−^, OH^−^) coming from the electrolyte. As a result, an oxide layer begins to form on the surface of the metal. Concurrently, the migration of these ions continues through the oxide developed, enabling the growth of the anodic layer at both the metal/oxide and oxide/electrolyte interface [4]. Their structures and chemical properties depend on several process parameters, such as the electrolyte composition, applied stimulus (potential or current density), and anodizing time [5].

Although anodizing has been mainly studied in valve metals [6,7,8,9], the growth of anodic oxide layers in iron and steel-based materials is becoming important for applications in photocatalysis [10,11], biomedical [12], and corrosion resistance [13,14] after demonstrating a high mechanical strength, large specific surface area, and even improved electron mobility.

In corrosion concerns, the functionality and durability of materials are threatened by the decline of the mechanical properties that can lead to premature failure of the parts. Stainless steel (SS) has acceptable anticorrosive properties, but its efficiency is limited when subjected to aggressive aqueous environments with chloride ions. These ions are accountable for breaking the native/passive layer and causing localized corrosion [15]. Numerous methods have already been employed to minimize SS corrosion, based primarily on the use of a barrier layer between the surface and the surrounding environment [16,17,18,19]. However, rapidly fabricating the passive film with a suitable thickness and making the process economically and industrially profitable are still the challenges. For these reasons, the anodizing process is considered one of the best alternatives.

Regarding the anodic layers on SS, Kure et al. [20] reported the first formation of nanoporous oxide layers on 304 SS using an organic solution that contained a mixture of H_2_O and NH_4_F. The importance of the H_2_O concentration in the organic electrolyte was explained in a later study by Klimas et al. [21]. They found that the anodic films were not formed in the glycerol solution when the H_2_O concentration exceeded 3 vol% due to the anodic layers presenting higher chemical dissolution rate than oxide formation rate. Since then, the influence of some parameters on the formation of anodic layers on SS has been reported [22,23,24,25], while only a few researchers have analyzed its electrochemical behavior against corrosion [14,26,27]. 

In all these studies, the addition of NH_4_F to the electrolyte was a key factor for ensuring the growth of porous and nanotube-like anodic layers. Unfortunately, the NH_4_F could cause significant environmental and health damage as it is a hazardous reagent. Therefore, finding new sustainable conditions for anodizing of SS should be one of the most important goals in this area. 

Recently, the use of silicate, aluminate, and phosphate-based electrolytes with high anodizing potential has successfully produced thick films on SS. Andrei et al. [28] obtained layers composed of Al_2_O_3_, FeAl_2_O_4_, and Fe_2_O_3_ triggered by the decomposition of the electrolyte containing 0.3 M NaAlO_2_. On the other hand, Malinovschi et al. [29] developed uneven, porous films in an electrolyte containing Na_2_SiO_3_ and Na_2_CO_3_, formed mainly by amorphous SiO_2_, Fe_2_O_3_, and Fe_3_O_4_. However, Yang et al. [30] compared aluminate and silicate electrolytes for growing anodic films in low carbon steel and evaluated their corrosion resistance. In this study, both anodic coatings showed a more positive corrosion potential and a lower corrosion current density than the substrate in 3.5% NaCl; but the layer developed in the aluminate condition (10 g/L NaAlO_2_ and 1.5 g/L NaH_2_PO_4_⦁2H_2_O) had a denser structure with better anti-corrosion properties.

Since the previous studies were led using aqueous electrolytes and high anodizing potentials, this work is focused on obtaining new conditions for anodizing SS (type-304L) using fluorine-free organic electrolytes containing NaAlO_2_ or Na_2_SiO_3_. In this first approach, the influence of the H_2_O concentration in the anodizing electrolyte was studied and the electrochemical behavior of the fabricated anodic films was evaluated by potentiodynamic polarization tests.

## 2. Materials and Methods

### 2.1. Samples Preparation

A 22 mm diameter rod of commercial grade 304L SS supplied by Jay Steel Corporation, Mumbai, India, was cut into 5 mm thick disk samples. The elemental composition of 304L SS alloy was: 18.3 wt.% Cr, 8.11 wt.% Ni, 1.52 wt.% Mn, 0.27 wt.% Si, and balance of Fe. Before anodizing, the samples were manually polished using successive grades of SiC paper to 2000 grit, rinsed and cleaned with ethanol and distilled water, then dried in the air stream.

### 2.2. Anodizing Conditions

The anodizing was carried out using a two-electrode cell with the 304L SS sample as anode and a platinum electrode as a cathode located at a distance of ~15 mm from each other. A freshly prepared electrolyte was used for each treatment, maintaining a solution volume/sample area ratio of 38 mL/cm^2^. The electrolytes used were organic glycerol-based solutions containing NaAlO_2_ or Na_2_SiO_3_ and different H_2_O concentrations, as outlined in Table 1. In addition, the treatments were carried out at 25 °C under galvanostatic conditions with three different values of current density (i) and anodizing time (Table 1). 

The voltage–time curves of the above anodizing treatments that follow a similar behavior to others carried out under the same conditions are annexed in the Supplementary Material. After anodizing treatment, the samples were rinsed with distilled water and ethanol to remove the remaining ions from the solution, then dried in an airstream.

### 2.3. Anodic Layers Characterization

The morphological characterization of the anodic layers was examined by field emission gun scanning electron microscopy (FEG–SEM) using a Hitachi S-4800 and equipped with energy-dispersive X-ray spectroscopy (EDX, Manufactured by Hitachi Ltd, Tokyo, Japan), which operated at 15 keV for EDX analysis and 7 keV for secondary electron imaging. Raman spectra were recorded in a Thermo Scientific DRX Raman (Manufactured by Thermo Fisher Scientific, Waltham, MA, USA) with a HeNe gas laser (λ = 633 nm) and power of 2 mW. Each spectrum was recorded by an accumulation of 10–50 scans in a range of 50–1500 cm^−1^. Chemical surface composition was analyzed by X-ray photoelectron spectroscopy using a Thermo Scientific K-Alpha (Manufactured by Thermo Fisher Scientific) equipped with Al Kα radiation (hν = 1486.6 eV) and operated at 12 kV, and 40 W. XPS spectra were obtained using the small windows mode with 0.1 eV/step and pass energy of 50 eV. Binding energies of the photoelectrons were calibrated using a contaminant carbon peak (C1s at 284.8 eV), and the spectra were analyzed with the Thermo Scientific Advantage data system (Manufactured by Thermo Fisher Scientific) for surface analysis. The X-ray diffraction (XRD) pattern was obtained using a Panalytical Xpert Pro diffractometer (Manufactured by Malvern Panalytical a spectrics company, Malvern, UK) with Cu-Kα (1.5406 Å), acceleration voltage of 45 kV, and working current of 40 mA.

On the other hand, corrosion behavior was evaluated by potentiodynamic polarization using a Gamry Reference 600 potentiostat (Manufactured by GAMRY Instruments, Warminster, PA, USA). Electrochemical measurements were carried out in duplicate (less in aluminate solutions) using a conventional three-electrode cell with a 0.3 M NaCl solution at 25 °C. The working electrodes were the 304L SS samples, the reference electrode was a Ag/AgCl (3 M KCl) electrode, and the counter electrode was a platinum wire. Before the tests, the samples were masked with Red Stop-Off Lacquer (product code: 166054, MacDermid Española S.A, Barcelona, Spain), leaving exposed only the area to be evaluated (~0.5 cm^2^). The potentiodynamic curves were conducted at a scan rate of 0.16 mV/s. The potential scan was performed in the anodic direction from a cathodic potential value of 300 mV with respect to the corrosion potential to 1 V with respect to the reference electrode or until the current density had reached a limit value of 0.25 mA/cm^2^.

## 3. Results and Discussion

### 3.1. Characterization of the Anodic Layers Obtained in NaAlO_2_ Electrolytes

**Figure 1:**
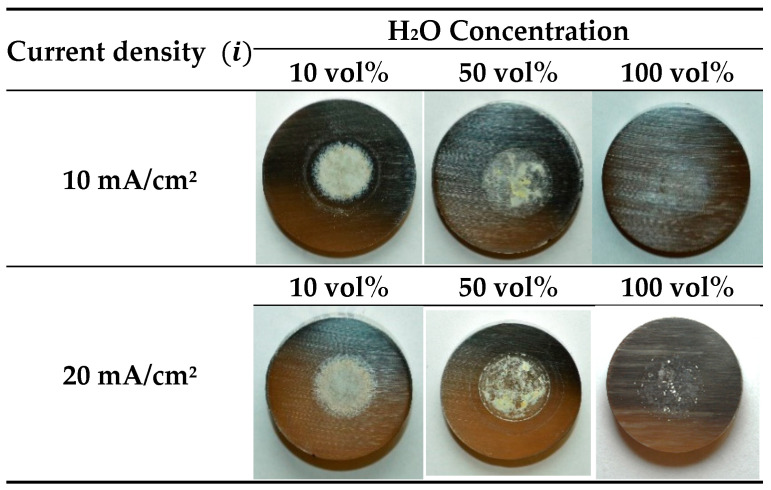
Photographs of 304L SS after anodizing in glycerol electrolyte containing 0.3 M NaAlO_2_ and different H_2_O concentrations (10–50–100 vol%) for 30 min at 10 and 20 mA/cm^2^.

The voltage–time curves and the macroscopic images of the surface appearance after anodizing processes in NaAlO_2_ electrolytes with different concentrations of H_2_O are shown in Figure S1 and Figure 1, respectively. It can clearly be seen that the H_2_O content has an important influence on the growth of the anodic layers since more compact and adherent oxide films were obtained when its percentage decreases. Indeed, treatments with percentages of H_2_O ≥ 50 vol% presented a loose layer on their surface, while the oxide layer formed in 10 vol% was more uniform and coarser. Additionally, no significant effect was observed on the layers for anodizing using different current densities (10–20 mA/cm^2^).

**Figure 2:**
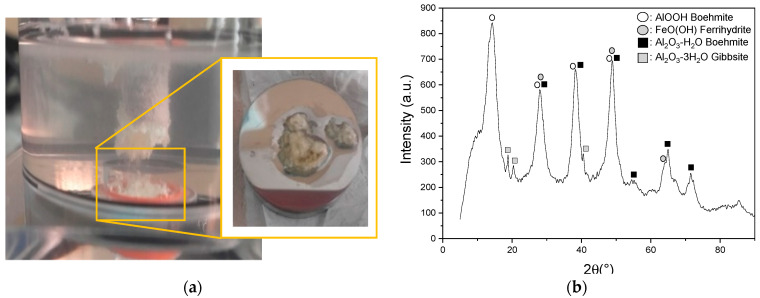
(**a**) Precipitate formed on the 304L SS samples during NaAlO_2_ treatments, and (**b**) XRD pattern of precipitate from (**a**).

On the other hand, a lumpy precipitate was generated under all anodizing conditions, perhaps due to the decomposition and agglomeration of the aluminate compounds within the electrolyte (Figure 2a). Such precipitate could contain relatively unstable aluminate ions that originate from the dissociation of NaAlO_2_ in the electrolyte. Previous studies by Carreira et al. [31] concluded through the Raman and IR techniques that there are two species of aluminate ions: (1) Al(OH)_4_^−^, predominant in a pH range between 8–12, and (2) AlO_2_^−^, abundant in solutions with a pH above 12.5. However, Yerokhin et al. [32] affirmed that the main aluminum species in concentrated NaAlO_2_ solutions is Al(OH)_4_^−^. Considering this, Cheng et al. [33] associated the hydrolysis of NaAlO_2_ and its decomposition into aluminate ions during micro-arcs oxidation (MAO) treatments with Equation (1) and Equation (2). These authors affirmed that if the electrolyte is decomposed, blocks of precipitated Al(OH)_3_ can be easily found in the bottom of the reaction vessel [34], similar to those obtained in this study. Moreover, it is well known that Al(OH)_3_ is transformed into AlOOH (Equation (3)) and then converted to Al_2_O_3_ (Equation (4)) [35]. The XRD pattern confirmed that the anodic layer grown in NaAlO_2_ contained a mixture of Al_2_O_3_, AlOOH, and FeOOH but exhibited limited adhesion to the 304L SS (Figure 2b).

(1) $${\mathrm{NaAlO}}_{2}+2H_{2}O\to \mathrm{Al}{\left(\mathrm{OH}\right)}_{4}{}^{-}+{\mathrm{Na}}^{+}$$

(2) $$\mathrm{Al}{\left(\mathrm{OH}\right)}_{4}{}^{-}\to \mathrm{Al}{\left(\mathrm{OH}\right)}_{3}+{\mathrm{OH}}^{-}$$

(3) $$\mathrm{Al}{\left(\mathrm{OH}\right)}_{3}\to \mathrm{AlOOH}+H_{2}O$$

(4) $$2\mathrm{AlOOH}\to {\mathrm{Al}}_{2}O_{3}+H_{2}O$$

**Figure 3:**
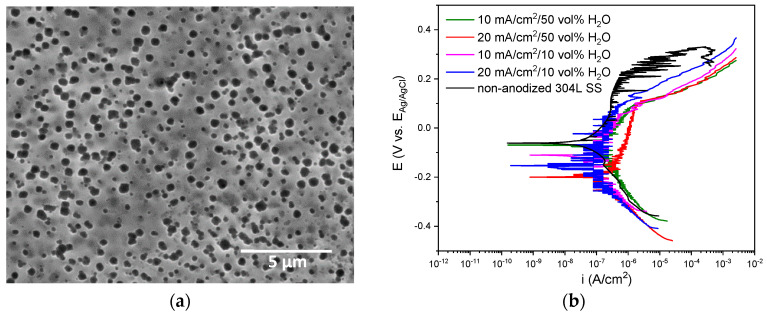
(**a**) SEM image of the anodic layer grown in a glycerol, 0.3 M NaAlO_2_ and 10 vol% H_2_O for 30 min at 20 mA/cm^2^. (**b**) Potentiodynamic polarization curves for anodic layers grown in glycerol electrolyte containing 0.3 M NaAlO_2_.

Regarding the SEM analysis, the only adherent oxide layer fabricated in this type of electrolyte is presented in Figure 3a. The result corroborated the lack of surface uniformity mentioned above by observing the coated and devoid areas of the anodic layer. These zones can result from the evolution of fine bubbles formed as a result of the release of oxygen during the anodizing process, hindering contact of the surface with the electrolyte and, therefore, the growth of the layer in those areas.

The electrochemical stability of the anodic films was determined by potentiodynamic polarization curves shown in Figure 3b. The non-anodized 304L SS substrate evidences a corrosion potential (E_corr_) of −63.2 ± 3.1 mV vs. Ag/AgCl (3 M KCl), a pitting potential (E_pitt_) of 271.7 ± 36.3.6 mV vs. Ag/AgCl (3 M KCl) and a passive current density (i_pass_) of 7.75 × 10^−7^ ± 8.00 × 10^−8^ A/cm^2^. In contrast, the anodic branch of the film at 20 mA/cm^2^ and 50 vol% H_2_O describes a more active behavior than the non-anodized 304L SS, while the other treatments show a similar electrochemical behavior than the substrate with i_pass_ in the same range of magnitude, but with a smaller passive branch length. In general, all polarization curves move towards negative potentials, both E_corr_ and E_pitt_. The E_corr_ for the anodized ones varies between ~−199.1 and ~−68.3 mV vs. Ag/AgCl (3 M KCl), while the E_pitt_ were between ~51.3 and ~98.1 mV vs. Ag/AgCl (3 M KCl). Moreover, the i_pass_ of the anodic layers varies between ~1.86 × 10^−7^ and ~9.86 × 10^−7^ A/cm^2^. These results indicate that the anodic layers exhibit a higher susceptibility to localized corrosion than the non-anodized specimen but have a similar corrosion rate.

This behavior can be attributed to the cavities observed in the SEM images and the poor adhesion of the layers in most of the conditions evaluated with this electrolyte. According to Yang et al. [36], a hole that permits direct contact between the substrate and the electrolyte decreases the anticorrosive performance since it acts as a channel, allowing a pitting corrosion mechanism to develop. In addition, the electrochemical behavior of the anodic layers can also be associated with the absence of a stable barrier layer in the metal/oxide interface, as mentioned by Andrei et al. [28,37]. They advised the formation of a barrier layer of iron oxide without Ni and Cr before the advanced anodizing treatments (MAO) to obtain coatings on austenitic stainless steel using aqueous solutions of NaAlO_2_. Thus, it is likely to be required to get effective results in conventional anodizing as well.

### 3.2. Characterization of the Anodic Layers Obtained in Na_2_SiO_3_ Electrolytes

**Figure 4:**
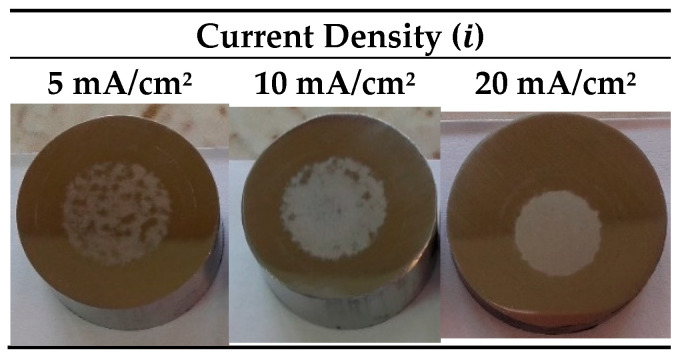
Photographs of 304L SS after anodizing in glycerol electrolyte containing 0.1 M Na_2_SiO_3_ and 2.5 vol% H_2_O for 15 min at 5–10–20 mA/cm^2^.

The voltage–time curves and the macroscopic images of the surface appearance of anodic layers grown at different current densities in a glycerol solution containing 0.1 M Na_2_SiO_3_ and 2.5 vol% H_2_O are shown in Figure S2 and Figure 4, respectively.

**Figure 5:**
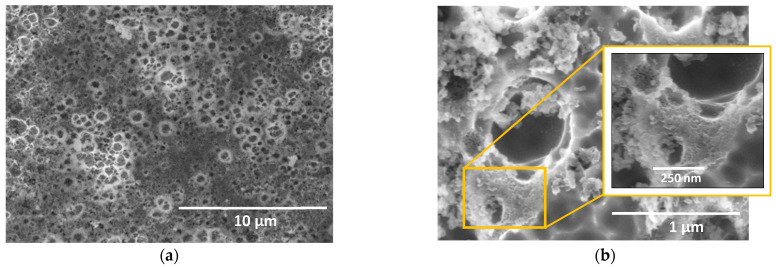
SEM images of the anodic layers grown in glycerol, 0.1 M Na_2_SiO_3_, and 2.5 vol% H_2_O for 15 min at 20 mA/cm^2^.

The development of a compact layer was favored just as the current density increased. For the anodizing at 20 mA/cm^2^, a uniform and adherent layer was observed on the surface of the 304L SS. In this case, SEM images (Figure 5a) also reveal the formation of small craters in the oxide layer, possibly originated by eruptions during the process. However, unlike the cavities obtained in NaAlO_2_, these craters showed an oxide film inside (Figure 5b). This outcome indicated that the formation of a barrier layer exists, which prevents direct contact of the metal with the surrounding medium (Figure 5b).

The EDS results (Figure S3) revealed that the anodic layer was composed of 4.28 wt.% C, 3.05 wt.% O, 0.65 wt.% Si, 17.84 wt.% Cr, 63.79 wt.% Fe, 8.23 wt.% Ni, and 2.16 wt.% Mn. The presence of C in the anodic layers is usually detected in anodizing with organic electrolytes due to its incorporation from the solution [15,20,21]. Moreover, the increase in Si content concerning the concentration of Si in 304L SS substrate can be attributed to the formation of a silica-based gel at the oxide/electrolyte interface that favors the mobility of ions such as SiO_3_^2−^, HSiO_3_^−^, Si^4+^, and retains them in its structure according to Mato et al. [38].

**Figure 6:**
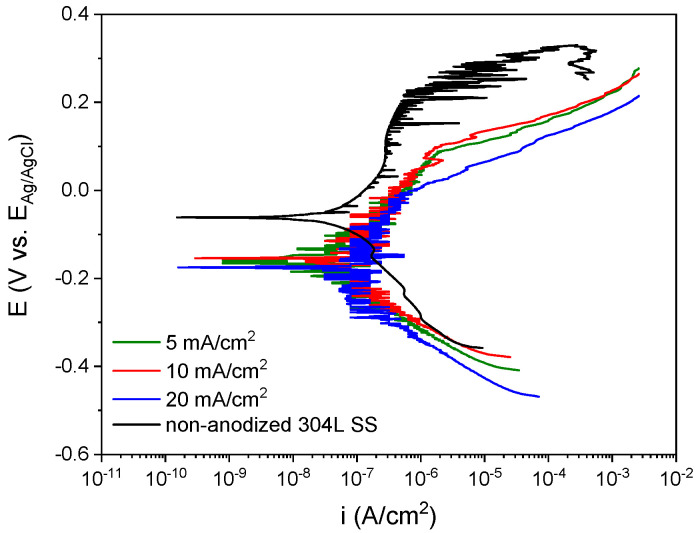
Potentiodynamic polarization curves for anodic layers grown in glycerol electrolyte containing 0.1 M Na_2_SiO_3_ and 2.5 vol% H_2_O.

The potentiodynamic polarization curves of each anodic layer are presented in Figure 6. The anodic layers showed similar behavior among them and disclosed similar values of E_corr_ around −198.4 ± 23.3 mV vs. Ag/AgCl (3 M KCl), E_pitt_ about 37.0 ± 21.9 mV vs. Ag/AgCl (3 M KCl), and i_pass_ around 3.12 × 10^−7^ ± 3.25 × 10^−8^ A/cm^2^. This implies that, regardless of the current density applied, the dissolution rate of the anodic layers in NaCl solution was similar to the non-anodized 304L SS. In addition, the corrosion behavior of the Na_2_SiO_3_ anodic layers resembled the NaAlO_2_ ones. Nevertheless, the decrease of the H_2_O concentration in the anodizing bath seems to produce a uniform anodic layer, possibly caused by the attenuation of the chemical dissolution mechanism in organic electrolytes as mentioned in the studies carried out by LaTempa et al. [39]. Likewise, Klimas et al. [21] pointed out that the growth of anodic layers on SS in organic electrolytes with fluorides is not possible when the H_2_O concentration exceeds 3 vol%; therefore, we decided to study the effect of the H_2_O concentration in the electrolyte containing Na_2_SiO_3_ for this work.

#### Influence of H_2_O Concentration in Electrolyte Containing Na_2_SiO_3_

**Figure 7:**
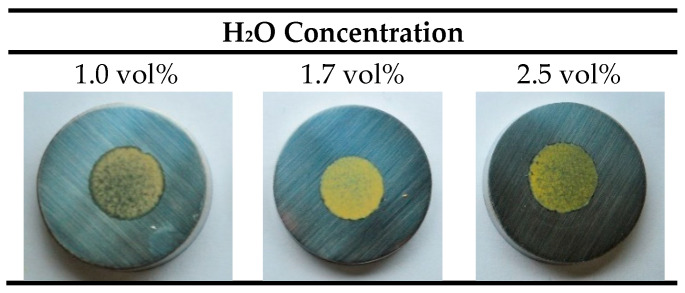
Photographs of 304L SS after anodizing in glycerol electrolyte containing 0.05 M Na_2_SiO_3_ and different H_2_O concentration (1.0–1.7–2.5 vol%) for 6 min at 20 mA/cm^2^.

The voltage–time curves and the surface appearance of the 304L SS samples after anodizing in the Na_2_SiO_3_ solution with different H_2_O concentrations and a constant current density of 20 mA/cm^2^ are shown in Figure S4 and Figure 7, respectively. It can be seen that the H_2_O concentration plays an important role in the uniformity of the layers. The layer turned out to be more compact when H_2_O concentration was 1.7 vol%, while the surface coverage decreased for H_2_O concentrations of 1.0. vol% and 2.5 vol%.

**Figure 8:**
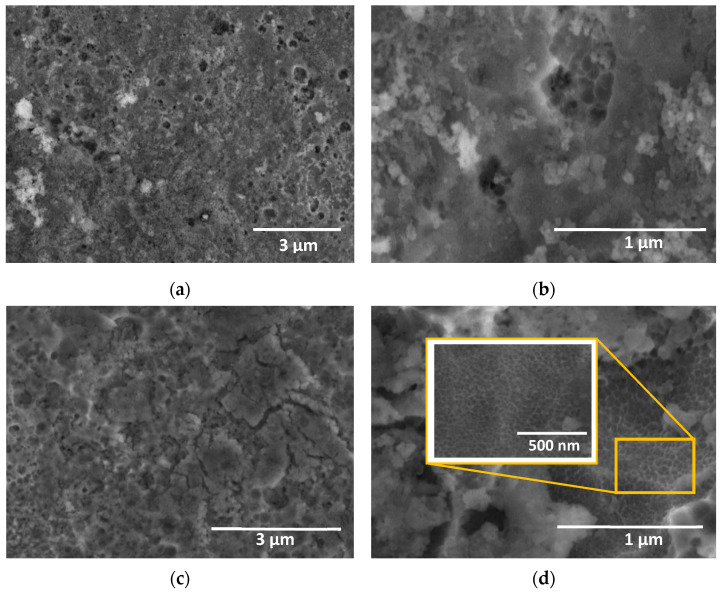

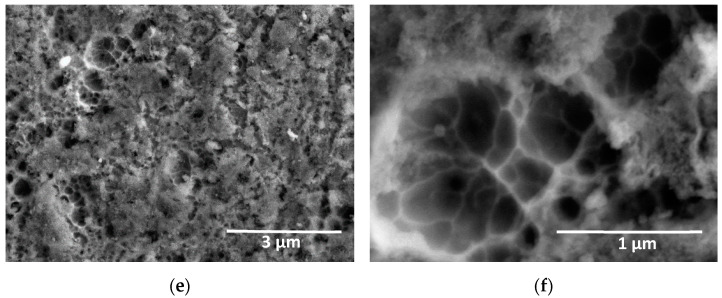
SEM images of the anodic layers grown in a glycerol, 0.05 M Na_2_SiO_3_ with H_2_O concentration of (**a**,**b**) 1.0, (**c**,**d**) 1.7, and (**e**,**f**) 2.5 vol% for 6 min at 20 mA/cm^2^.

SEM images showed that the anodic layer grown at the lowest concentration of H_2_O presented a smooth surface with fewer defects (Figure 8a,b). However, rupture of the layers was observed when the concentration increased from 1.0 vol% to 1.7 vol% H_2_O, revealing the presence of apparent nanopores with small craters inside them (Figure 8c,d). Instead, micrometer-sized craters were formed in the anodic films fabricated at the higher H_2_O concentration (Figure 8e,f). This oxide film is similar to those described by Asoh et al. [12] in 304 SS using a H_2_SO_4_ and H_2_O_2_ containing electrolyte. In addition, the EDS analysis summarized in Figure S5 and Table 2 confirmed the presence of silicon in the oxide layer, probably coming from both the substrate and the electrolyte.

Therefore, the H_2_O concentration in the electrolyte resulted in a key factor for obtaining uniform films in Na_2_SiO_3_-electrolyte. Considering this, the results in Figure 7 and Figure 8 confirmed that the H_2_O concentration of 1.7 vol% was the ideal condition to balance the mechanisms of formation and dissolution of the anodic layer in this type of electrolyte. 

**Figure 9:**
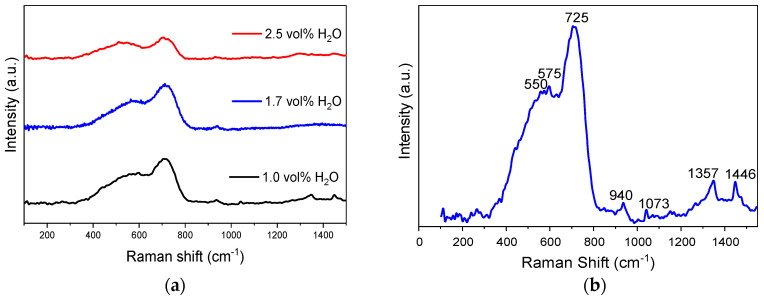
Raman spectra of the anodic layers grown in glycerol, 0.05 M Na_2_SiO_3_ with H_2_O concentrations (**a**) from 1.0 to 2.5 vol%, and (**b**) 1.7 vol% for 6 min at 20 mA/cm^2^.

Regarding the Raman analysis, all the spectra of the anodic layers grown at 20 mA/cm^2^ in the Na_2_SiO_3_-electrolyte with different H_2_O concentrations are presented in Figure 9a. It is observed that the anodizing treatments did not show important changes between them, so the spectrum of the oxide layer obtained in 1.7 vol% H_2_O is analyzed as an example in Figure 9b.

The higher intensity bands identified at 725 cm^−1^ (Fe^3+^) and 550 cm^−1^ (Fe^2+^) were associated with the Fe–O stretching mode. This bond occurs in compounds such as magnetite (Fe_3_O_4_), maghemite (γ-Fe_2_O_3_), and goethite (α-FeOOH) [40]. The low intensity bands at 1357 and 1446 cm^−1^ corresponded only to the α-FeOOH and γ-Fe_2_O_3_ species, respectively, indicating their presence in the anodic layer [41]. On the other hand, the presence of an intense band at 550 cm^−1^ could be assigned to Ni–O stretching modes of Ni(OH)_2_ [42], which overlaps with iron oxides peaks. In addition, the bands at 575 and 497 cm^−1^ indicated that the formation of mixed oxides of Fe and Ni is possible [43]. The band at 939.6 cm^−1^ represented the Cr=O stretching mode [44], and it is related to the presence of chromium oxide in the anodic layers. Raman weak band around 1070 cm^−1^ should be assigned to the vibrational mode of the Si-O group, suggesting the formation of SiO_2_ in the anodic layers [45].

**Figure 10:**
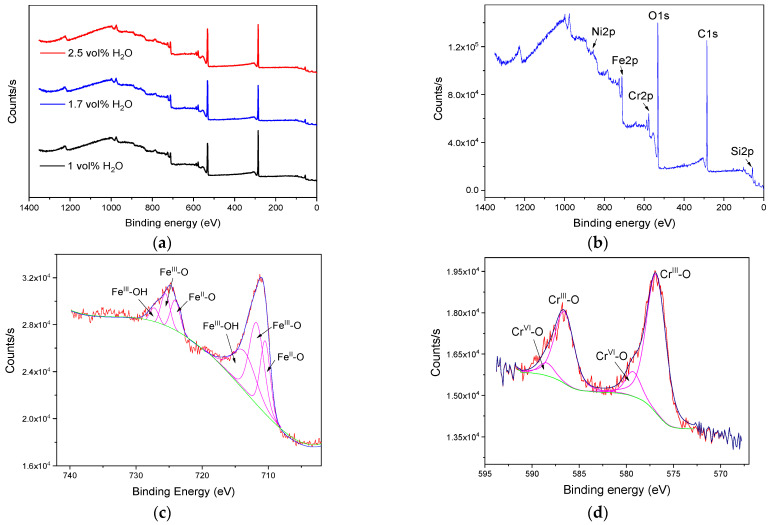

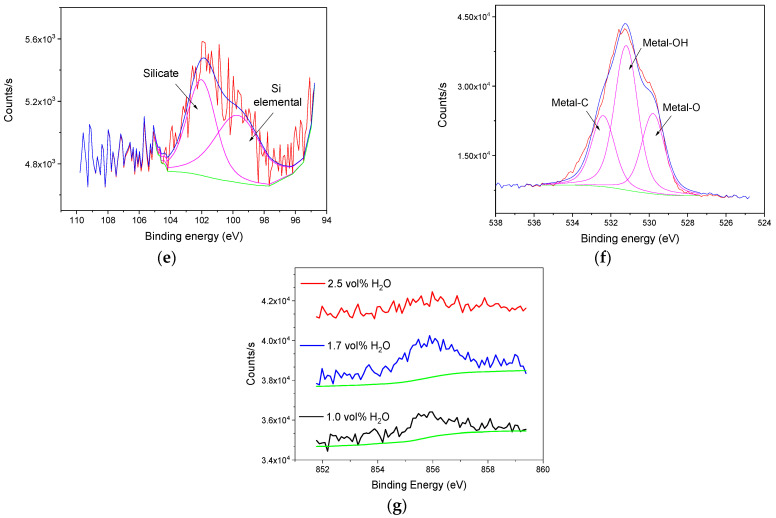
XPS spectra of the anodic layers grown in glycerol, 0.05 M Na_2_SiO_3_ with H_2_O concentration (**a**) from 1.0 to 2.5 vol%, and (**b**) 1.7 vol% for 6 min at 20 mA/cm^2^. Deconvolutions plots of the (**c**) Fe2p, (**d**) Cr2p, (**e**) Si2p, (**f**) O1s, and (**g**) Ni2p scan of the anodic layers grown in glycerol, 0.05 M Na_2_SiO_3_ and 1.7 vol% H_2_O.

XPS analysis was performed to provide information about the chemical composition of the layers grown in the Na_2_SiO_3_ electrolytes with different H_2_O concentrations (Figure 10a). In all samples, the characteristic peaks of Fe, Cr, Si, O, and C were identified in the survey spectra. Only the layers that grew using H_2_O concentrations ≤1.7 vol% showed the Ni peak, which became more noticeable when H_2_O concentration was 1.7 vol% (Figure 10b,g).

The high-resolution window of Fe2p, Figure 10c, allowed identification of the presence of the Fe^II^-O bond at 710.7 eV, suggesting the formation of FeO or Fe_3_O_4_. Furthermore, the Fe^III^-O and Fe^III^-OH bonds at binding energies of 712.11 and 713.8 eV, respectively, are related to the possible presence of Fe_2_O_3_ and FeO(OH) in the layer. These results were in agreement with results published by Pawlik et al. [22] in anodizing on pure iron using organic electrolytes with fluorides.

The high-resolution window of Cr2p, Figure 10d, evidenced two peaks at binding energies of 576.6 and 578.8 eV, indicating the presence of both Cr^III^ (78%) and Cr^VI^ (22%) in the oxide, as hydroxide or oxyhydroxide form [20]. Furthermore, the incorporation of silicate species in the anodic layer was observed in the deconvolution of the Si2p peak in 101.4 eV, Figure 10e. This indicated the presence of Si (23%) associated at a binding energy of 98.3 eV and silicate (77%), which peak appears at 101.7 eV. These results are analogous to those reported by Hsiao et al. [46] in anodizing treatments on Mg alloys using electrolytes containing Na_2_SiO_3_. Finally, the high-resolution window of O1s, Figure 10f, revealed the presence of three peaks: the first one at 529.8 eV (22%) ascribed to the O–Metal bond that suggests the formation of Fe and Cr oxides; the second one at 531.7 eV (48%), associated to hydroxides of Fe and Cr; and the third one at 532.4 eV (30%) assigned to O–C bond [21]. Therefore, the above results indicated that the anodic layer was mainly composed of oxides, hydroxides, and oxyhydroxides of Fe, Cr, Si, and carbonates of Fe. Furthermore, it may also contain Ni species that, due to the detection limit of the equipment (0.2 at.%), was not possible to carry out the deconvolution of the Ni peak in the high-resolution window and determine its concentration in the anodic layers (Figure 10g).

**Figure 11:**
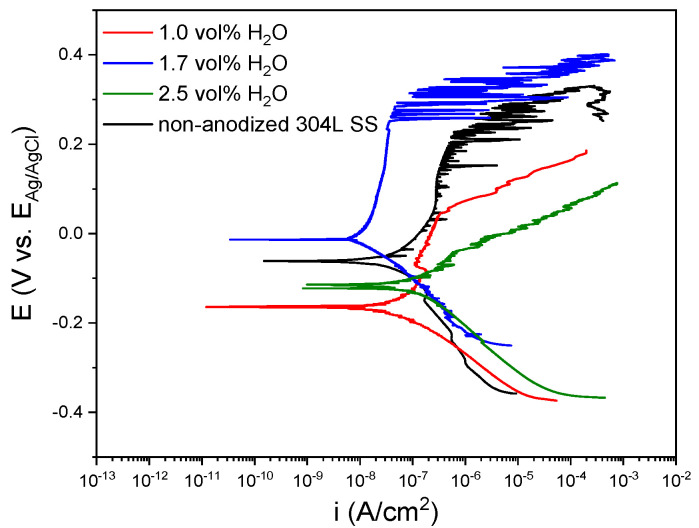
Potentiodynamic polarization curves for anodic layers grown in glycerol electrolyte containing 0.05 M Na_2_SiO_3_ and different H_2_O concentrations.

The potentiodynamic polarization curves of the oxide layers fabricated in a Na_2_SiO_3_ electrolyte with different H_2_O concentrations are presented in Figure 11. Changes in the polarization curves of the layers were shown regarding the non-anodized 304L SS. When the H_2_O concentration increased from 1.0 vol% to 1.7 vol% the i_pass_ values varied from 3.85 × 10^−8^ ± 1.17 × 10^−8^ A/cm^2^ to 9.45 × 10^−8^ ± 2.55 × 10^−8^ the E_corr_ shifted from –162.35 ± 1.75 to −18.92 ± 5.41 mV vs. Ag/AgCl (3M KCl) and the E_pitt_ changed from 42.0 ± 2.0 to 243.3 ± 6.5 mV vs. Ag/AgCl (3 M KCl). These findings indicated a better electrochemical response against corrosion for the anodic layer grown with 1.7 vol% H_2_O concentration. Moreover, it could be seen that the electrochemical behavior of the anodic layers worsened when H_2_O percentage increased from 1.7 vol% to 2.5 vol%, reaching values of 2.98 × 10^−7^ ± 2.70 × 10^−8^ A/cm^2^ for i_corr_, −117.6 ± 1.1 mV vs. Ag/AgCl (3 M KCl) for E_corr_, and −57.5 ± 18.8 mV vs. Ag/AgCl (3 M KCl) for E_pitt_. These results imply that the anodic layers with better protective properties were obtained for 1.7 vol% H_2_O concentration in the electrolyte, improving the corrosion resistance of the non-anodized 304L SS substrate by approximately one order of magnitude.

**Figure 12:**
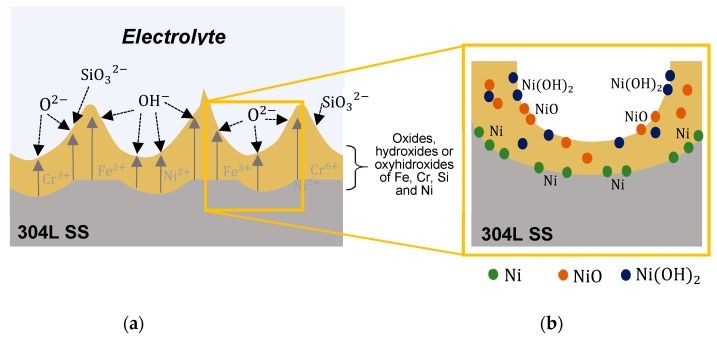
(**a**) Schematic illustration of the mechanism during the anodizing process. (**b**) Distribution of Ni in the anodic layer.

This better corrosion behavior for anodic layers grown in the bath containing 1.7 vol% H_2_O may be associated with the presence of Ni in the oxide as it was found in the XPS spectra. It is commonly accepted that, in addition to Cr, Ni plays an important role in austenitic SS to improve the corrosion resistance [47], while for steels with a high Ni content, this element is found in the native passive layer [48]. Figure 12 shows a schematic representation of Ni incorporation in the anodic layer during anodizing process.

Firstly, there is an anion migration (O_2_^−^, OH^−^, SiO_3_^2−^) from the electrolyte towards the SS interface as well as an ejection of cations from the material into the electrolyte triggered by the action of the electric field of the anodizing process (Figure 12a). Secondly, such ion migration outwards could distribute Ni into the anodic film as follows: (1) Ni accumulation in the oxide/metal interface in metallic form and (2) Ni presence in the anodic layer as Ni oxide/hydroxide compounds (Figure 12b). The first possibility is reported in the literature by Olsson et al. [49], who explained that the less active potential of Ni favors preferential oxidation of Fe and Cr. Therefore, the anodic layer is mainly composed of compounds of Fe and Cr while a metallic Ni enrichment is generated in the oxide/metal interface that delays the dissolution of the material. The second possibility is a consequence of the oxidation of Ni cations that are eluted outside the surface during ionic migration, which allows the formation of Ni compounds such as NiO and Ni(OH)_2_ [21].

## 4. Conclusions

Anodic layers were formed by a galvanostatic conventional anodizing process in fluoride-free electrolytes on 304L SS. The samples anodized in a based glycerol electrolyte with 0.3 M NaAlO_2_, and high H_2_O concentrations did not form uniform and protective oxide layers. In contrast, it was possible to obtain compact anodic layers using a glycerol electrolyte containing low concentrations of Na_2_SiO_3_ and H_2_O. 

The characterization of the anodic layers obtained in Na_2_SiO_3_ solutions showed that they were mainly composed of oxides, hydroxides, and oxyhydroxides of Fe, Cr, and Si. However, XPS results showed the presence of Ni in the samples anodized at concentrations ≤1.7 vol% H_2_O. The presence of Ni influences the chemical stability, suggesting that its distribution within the anodic layer provides better corrosion behavior than 304 L SS.

Thus, we concluded that the most optimal condition of those evaluated in this study to enhance the behavior against corrosion was the anodizing in glycerol with 0.05 M Na_2_SiO_3_, and 1.7 vol% H_2_O for 6 min at 20 mA/cm^2^. This condition improved the electrochemical response one order of magnitude compared to the non-anodized 304L SS.

## Figures and Tables

**Table 1:** The chemical compositions of the electrolytes and the anodizing conditions used for the fabrication of the oxide layers on 304L SS.

Electrolyte		[ ] ^1^ H_2_O (vol%)	$i\;(\mathrm{mA}/{\mathrm{cm}}^{2})$				Time (min)
Glycerol	0.3 M NaAlO_2_	10	10		20		30
		50					
		100					
	0.1 M Na_2_SiO_3_	2.5	5	10		20	15
	0.05 M Na_2_SiO_3_	1.0	20				6
		1.7					
		2.5					

^1^ Concentration.

**Table 2:** EDS analysis of the 304L SS anodic layers from Figure 8.

Anodizing Conditions	[ ] ^1^ H_2_O (vol%)	Content of Elements (in wt.%)						
		C	O	Si	Cr	Mn	Fe	Ni
Glycerol—0.05 M Na_2_SiO_3_—20 mA/cm^2^—6 min	1.0	5.14	4.50	0.46	17.40	0.93	64.88	6.70
	1.7	4.99	4.65	0.52	17.14	1.43	63.90	7.37
	2.5	10.76	5.35	0.46	16.38	1.08	59.59	6.38

^1^ Concentration.

## Data Availability

This study did not report any data.

## Supplementary Materials

The following supporting information can be downloaded at: https://www.mdpi.com/article/10.3390/ma15051892/s1: Figure S1, Voltage-time curves of anodizing in glycerol electrolyte containing 0.3 M NaAlO_2_ and different H_2_O concentration (10–50–100 vol%) for 30 min at 10 and 20 mA/cm^2^; Figure S2, Voltage-time curves of anodizing in glycerol electrolyte containing 0.1 M Na_2_SiO_3_ and 2.5 vol% H_2_O for 15 min at 5–10–20 mA/cm^2^; Figure S3, EDS diagram of the anodic layers grown in glycerol, 0.1 M Na_2_SiO_3_ and 2.5 vol% H_2_O for 15 min at 20 mA/cm^2^; Figure S4, Voltage-time curves of the anodic layers grown in glycerol electrolyte containing 0.05 M Na_2_SiO_3_ and different H_2_O concentration (1.0–1.7–2.5 vol%) for 6 min at 20 mA/cm^2^; Figure S5, EDS diagrams of the anodic layers grown in a glycerol, 0.05 M Na_2_SiO_3_ with H_2_O concentrations of (a) 1.0, (b) 1.7, and (c) 2.5 vol% for 6 min at 20 mA/cm^2^.
