# Supplementary material for: Growth of Anodic Layers on 304L Stainless Steel Using Fluoride Free Electrolytes and Their Electrochemical Behavior in Chloride Solution
Source: Materials (Basel). 2022 Mar 3;15(5):1892. doi: 10.3390/ma15051892 (PMC8912120; doi:10.3390/ma15051892)
Supplement: Supplementary file 1 [file materials-15-01892-s001.zip › materials-1534325-supplementary.pdf]

Supplementary Material

Growth Of Anodic Layers On 304L Stainless Steel Using Fluoride Free Electrolytes And Their Electrochemical Behavior In Chloride Solution

Laura Patricia Domínguez-Jaimes [1], María A. Arenas [2], Ana Conde [2], B. Escobar [3], Anabel Álvarez-Méndez[1] and Juan Manuel Hernández-López [1,*]

[1] Universidad Autónoma de Nuevo León, Facultad de Ciencias Químicas, Ciudad Universitaria, Av. Universidad s/n. C. P. 66455, Nuevo León, México.

[2] Department of Surface Engineering Corrosion and Durability, National Center for Metallurgical Research, CENIM-CSIC, Avda. Gregorio del Amo, 8, 28040 Madrid, Spain.

[3] CONACYT, Centro de Investigación Científica de Yucatán, Mérida, Yucatán, 97302, México.

* Correspondence: juan.hernandezlz@uanl.edu.mx; Tel.: + 52-1-81-83294000 ext. 6349

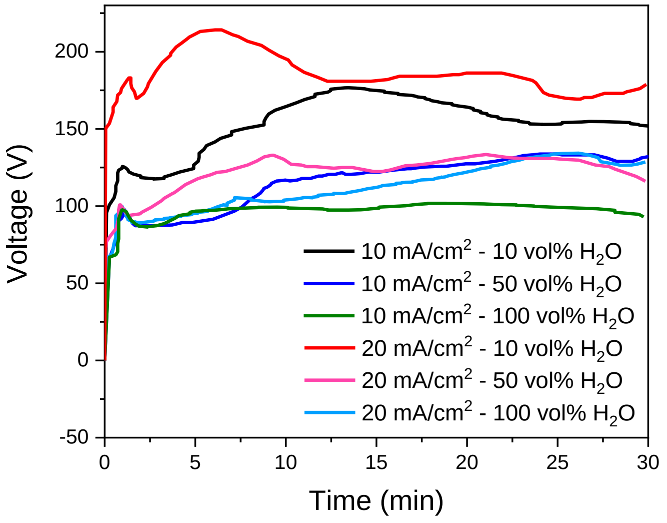

Figure S1. Voltage-time curves of the anodic layers grown in glycerol electrolyte containing 0.3 M $NaAlO_2$ and different H_2O concentrations (10-50-100 vol%) for 30 min at 10 and 20 mA/cm^2.

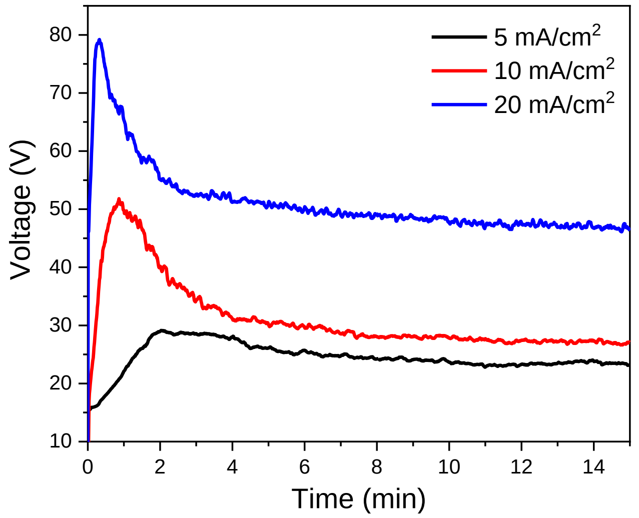

Figure S2. Voltage-time curves of the anodic layers grown in glycerol electrolyte containing 0.1 M Na_2SiO_3 and 2.5 vol% H_2O for 15 min at 5-10-20 mA/cm^2.

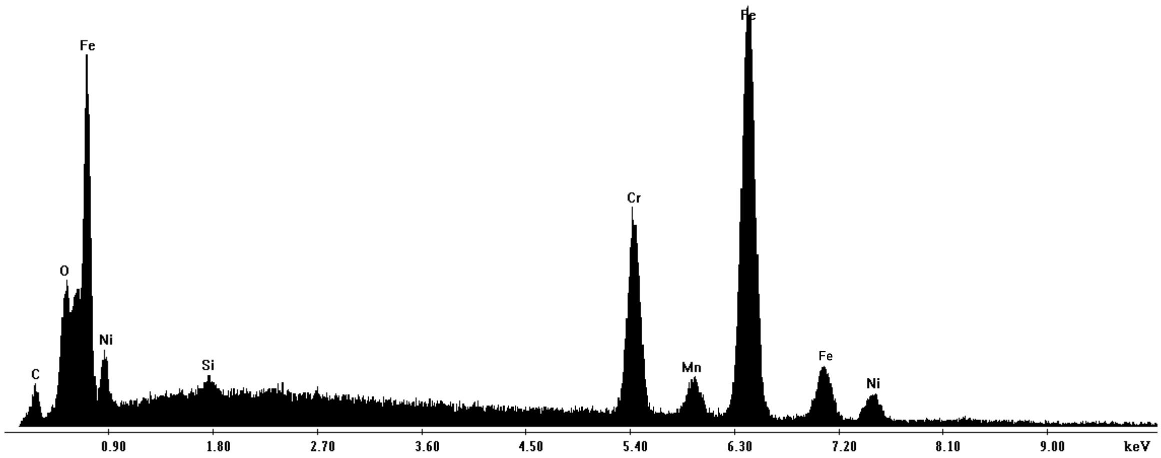

Figure S3. EDS diagram of the anodic layers grown in glycerol, 0.1 M Na_2SiO_3 and 2.5 vol% H_2O for 15 min at 20 mA/cm^2.

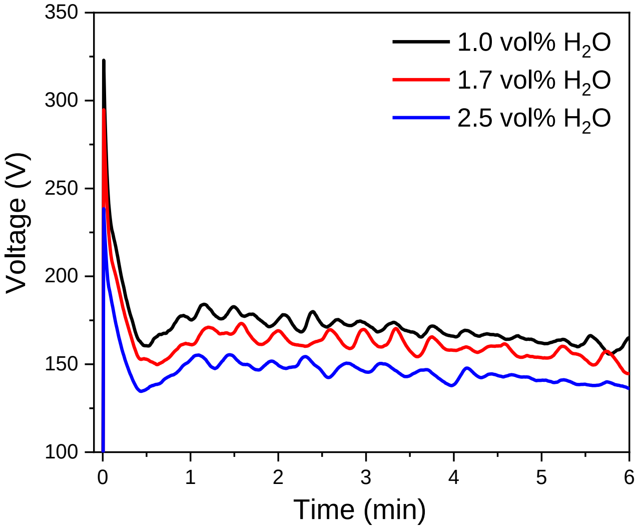

Figure S4. Voltage-time curves of the anodic layers grown in glycerol electrolyte containing 0.05 M Na_2SiO_3 and different H_2O concentration (1.0-1.7-2.5 vol%) for 6 min at 20 mA/cm^2.

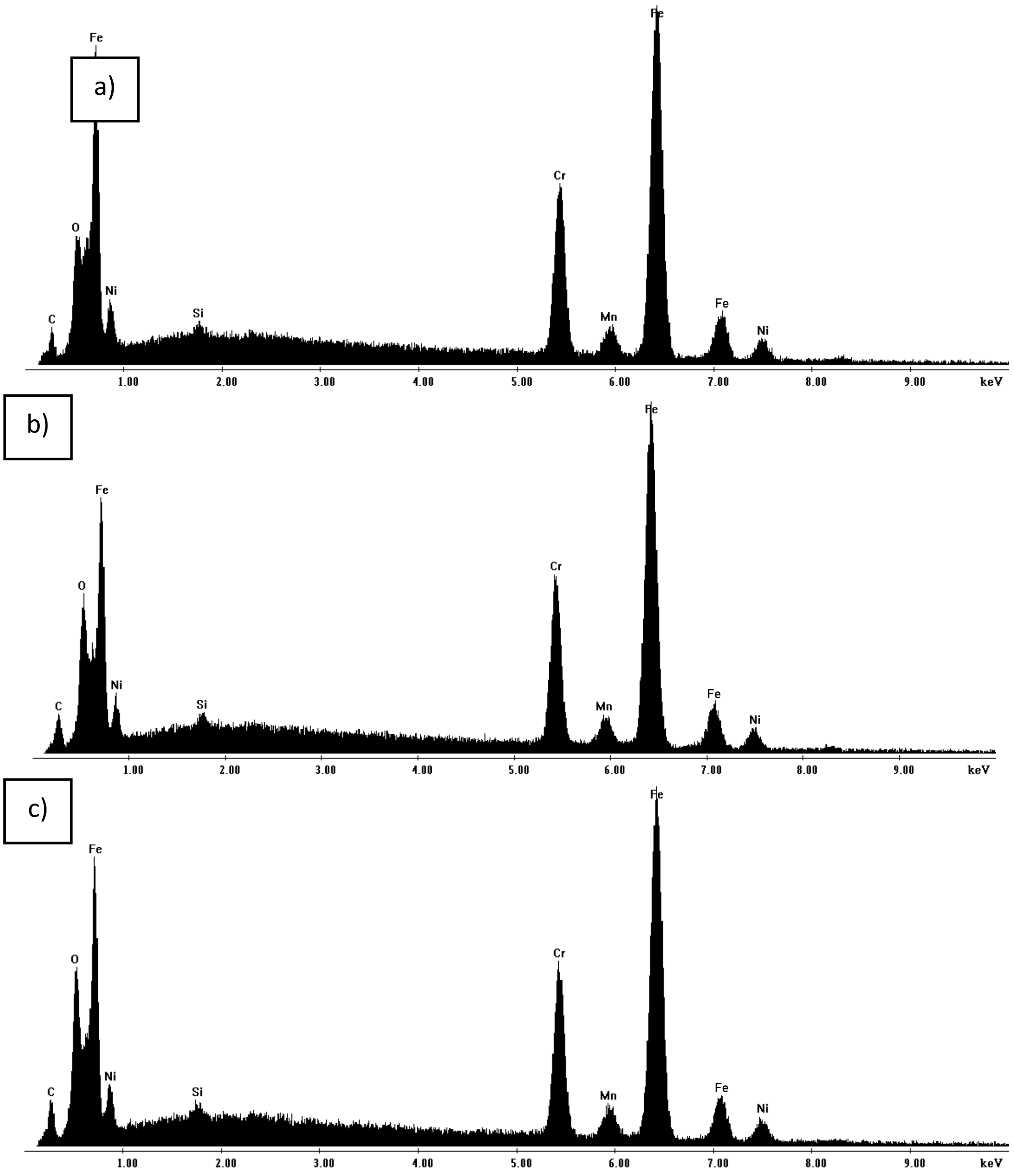

Figure S5. EDS diagrams of the anodic layers grown in a glycerol, 0.05 M Na_2SiO_3 with H_2O concentration of (**a**) 1.0, (**b**) 1.7, (**c**) 2.5 vol% for 6 min at 20 mA/cm^2.
